# Global epidemiology of *Cephalopina titillator* infestation in camels: a systematic review and meta-analysis (1980–2025)

**DOI:** 10.3389/fvets.2026.1863158

**Published:** 2026-07-08

**Authors:** Temesgen Mohammed, Aboma Zewude, Tsegaye Shamebo, Layaly Hamdan, Atef Oreiby, Hazim O. Khalifa, Gobena Ameni, Bojan Gajic

**Affiliations:** 1 Department of Veterinary Medicine, College of Agriculture and Veterinary Medicine, United Arab Emirates University, Al Ain, United Arab Emirates; 2 Institute of Public Health, College of Medicine and Health Sciences, United Arab Emirates University, Al Ain, United Arab Emirates; 3 Department of Health Sciences, College of Natural and Health Sciences, Zayed University, Academic City, Dubai, United Arab Emirates; 4 Department of Animal Medicine, Faculty of Veterinary Medicine, Kafrelsheikh University, Kafr El-Sheikh, Egypt; 5 Department of Pharmacology, Faculty of Veterinary Medicine, Kafrelsheikh University, Kafr El-Sheikh, Egypt

**Keywords:** camel, *Cephalopina titillator*, meta-analysis, myiasis, prevalence, systematic review

## Abstract

**Background:**

*Cephalopina titillator* (*C. titillator*) is the causative agent of camel nasopharyngeal myiasis and is widespread in camel-rearing regions worldwide, causing impaired health, reduced productivity, and economic losses. However, despite the veterinary importance of *C. titillator*, global epidemiological evidence remains limited and fragmented, and no comprehensive synthesis of prevalence data across camel-rearing regions has previously been conducted. The objective of this review was to estimate the global pooled prevalence of *C. titillator* infestation in camels. In addition, subgroup analyses were made to assess the effect of camel species, geographic region, and study period on the pooled prevalence of *C. titillator* infestation.

**Methods:**

The review was conducted in accordance with PRISMA 2020 guidelines. The review protocol was prospectively registered in the International Prospective Register of Systematic Reviews (PROSPERO) under registration number CRD420261344750. A literature search was conducted in multiple databases including PubMed/MEDLINE, Scopus, Web of Science (WoS), Google Scholar, and CAB Direct (Centre for Agriculture and Bioscience International database; CABI). Pooled prevalence was estimated by a random-effects meta-analysis using R software. Heterogeneity of the prevalence reports was assessed using Cochran’s Q and the I^2^ statistic. Funnel plot and Egger’s test were used for assessing study bias.

**Results:**

Forty-one epidemiological studies involving 18,959 camels from 11 countries were included in this review. The pooled prevalence of *C. titillator* infestation was 67% (95% CI: 59–75%). Heterogeneity among studies was very high (I^2^ = 99.1%, *p* < 0.001). Subgroup analyses revealed significant differences in pooled prevalence among study regions (*p* = 0.0016), whereas pooled prevalence was not significantly affected by study period or camel species (*p* > 0.05). Although the funnel plot showed slight asymmetry, Egger’s regression test did not indicate statistically significant small-study effects (*p* = 0.497).

**Conclusion:**

This meta-analysis demonstrated a high pooled prevalence of *C. titillator* infestation (67%; 95% CI: 59–75%) and revealed important geographical variation across camel-rearing countries, highlighting the substantial epidemiological burden of infestation and the need for region-specific surveillance and control strategies.

**Systematic review registration:**

PROSPERO, Registration No. CRD420261344750. Publicly accessible at: https://www.crd.york.ac.uk/prospero/display_record.php?RecordID=1344750.

## 1 Introduction

*Cephalopina titillator* infestation, commonly referred to as camel nasopharyngeal myiasis, is a parasitic disease affecting the upper respiratory tract of camels. *Cephalopina titillator* (Clark, 1797) belongs to the order Diptera, family Oestridae, and subfamily Oestrinae (1, 2). The larval stages of this oestrid fly develop within the nasal passages, paranasal sinuses, and pharyngeal cavities, where they attach to mucosal tissues and undergo progressive development (1, 2). The life cycle of the parasite begins when gravid female flies deposit first-stage larvae around the nostrils of camels. The larvae migrate through the nasal passages into the pharyngeal cavities, where they undergo successive developmental stages while remaining attached to the mucosal surfaces. Mature third-stage larvae are eventually expelled through sneezing, pupate in the soil, and subsequently emerge as adult flies, completing the life cycle (1, 3, 4).

The parasite is widely distributed across camel-rearing regions of Africa, the Middle East, and Asia and is recognized as a significant parasitic disease affecting camel health and productivity (1, 2). Recent epidemiological studies have shown that parasitic diseases remain major constraints to camel production globally, particularly in arid and semi-arid regions where camels play essential roles in food security, livelihood support, and climate-resilient livestock production. Gastrointestinal, ectoparasitic, and myiasis-associated infections collectively contribute to reduced productivity, impaired health, and substantial economic losses in camel-rearing systems (2, 5, 6).

Infestation with *C. titillator* is associated with a range of clinical manifestations, including nasal discharge, sneezing, respiratory distress, reduced feed intake, and general discomfort (7–9). The presence of larvae within the respiratory tract causes mechanical irritation and inflammatory responses that impair normal physiological function (2). In addition to mechanical damage, chronic infestation may induce marked mucosal inflammatory and immunological responses. Infested camels have been reported to exhibit eosinophilia, monocytosis, mucosal hypersecretion, and inflammatory tissue alterations associated with larval antigenic stimulation and excretory-secretory products (9–12). Larval attachment to the nasopharyngeal mucosa causes extensive irritation, epithelial damage, inflammatory lesions, and respiratory disorders that may predispose animals to secondary bacterial infections and chronic respiratory pathology (7, 13, 14). Furthermore, antigenic and inflammatory products released by the larvae may trigger hypersensitivity reactions and host immune dysregulation, contributing to disease progression and tissue injury (10, 11). In severe cases, tissue damage and larval migration may lead to complications such as secondary infections and, in some cases, neurological involvement due to larval migration (7, 9).

The impact of *C. titillator* infestation extends beyond clinical disease to include substantial production and economic consequences. Infested camels commonly exhibit reduced feed intake, weight loss, impaired body condition, decreased milk production, lower reproductive performance, and reduced market value (7, 8, 12). Severe infestations may impair respiratory function and working efficiency, thereby negatively affecting camel productivity and pastoral livelihoods in arid and semi-arid production systems (9, 15). Previous reports further indicate that chronic parasitic infestations in camels contribute considerably to economic losses through reduced meat and milk yield, increased management costs, poor body condition, and decreased reproductive performance in camel-dependent production systems (16–18).

Epidemiological studies conducted in different regions have reported considerable variation in the prevalence of *C. titillator* infestation. This variation is influenced by environmental factors such as temperature and humidity, seasonal patterns, host characteristics including age and body condition, and differences in husbandry systems (1, 17, 19). In addition, methodological differences between studies contribute to variability in reported prevalence. Most studies relied on postmortem larval detection in abattoirs, whereas others employed immunological methods such as ELISA, which may detect both current and past exposure and therefore yield different estimates (12, 20). These differences complicate direct comparison and interpretation of prevalence data across studies.

The available evidence on *C. titillator* infestation remains fragmented and largely region-specific, with most previous publications consisting of regional surveys, narrative reviews, or broader overviews of camel parasitic diseases rather than systematic quantitative syntheses specifically focused on *C. titillator* prevalence (2, 5). To date, no global systematic review and meta-analysis has comprehensively evaluated the pooled prevalence and epidemiological distribution of *C. titillator* infestation across camel-rearing regions. This lack of integrated evidence limits accurate assessment of the global burden of infestation and hinders identification of important epidemiological patterns and determinants.

Therefore, the present systematic review and meta-analysis was conducted to estimate the global pooled prevalence of *C. titillator* infestation in camels and to explore epidemiological variation according to geographical region, study period, and host species. We hypothesized that *C. titillator* infestation remains highly prevalent globally and that prevalence varies according to geographical region, study period, and camel species. By synthesizing available evidence, this study seeks to provide a clearer epidemiological baseline that may support evidence-based surveillance planning, region-specific parasite control strategies, improved epidemiological monitoring, and prioritization of future veterinary research and intervention programs in camel-rearing regions affected by this important parasitic disease.

## 2 Methods

### 2.1 Study design and protocol

This systematic review and meta-analysis was conducted in accordance with the Preferred Reporting Items for Systematic Reviews and Meta-Analyses (PRISMA 2020) guidelines. The review protocol was prospectively registered in the International Prospective Register of Systematic Reviews (PROSPERO) under registration number CRD420261344750 (21). The review was designed to estimate the pooled prevalence of *C. titillator* infestation in camels and to explore variation by geographical region, study period, and host species.

### 2.2 Literature search

A comprehensive literature search was performed in PubMed/MEDLINE, Scopus, Web of Science (WoS), Google Scholar, and CAB Direct (Centre for Agriculture and Bioscience International database; CABI) to identify relevant studies on *C. titillator* infestation in camels. Additional records were identified by manually screening the reference lists of retrieved articles. The search strategy combined keywords and synonyms using Boolean operators, including “*Cephalopina titillator*,” “camel,” “*Camelus dromedarius*,” “*Camelus bactrianus*,” “nasal bot fly,” “nasopharyngeal myiasis,” “myiasis,” and “prevalence.” Synonyms were combined using “OR,” and concepts were linked using “AND.” Studies published in English between 1980 and 2025 were considered. The search, screening, and data-handling procedures were developed in line with methodological guidance for systematic reviews of prevalence studies (22).

### 2.3 Eligibility criteria and data extraction

Studies were included if they: (i) involved camels (*Camelus dromedarius* or *Camelus bactrianus*), (ii) reported prevalence data for *C. titillator* infestation, and (iii) provided sufficient information to extract or calculate the number of positive animals and the total number examined (22). Review articles, case reports, experimental studies, conference abstracts, duplicate publications, and studies lacking extractable numerator–denominator data were excluded. Studies that did not specifically report *C. titillator* infestation in camels were also excluded.

All identified records were imported into covidence systematic review software (Veritas Health Innovation, Melbourne, Australia), and duplicates were removed. Two reviewers independently screened titles and abstracts for relevance, followed by full-text assessment of potentially eligible studies. Any disagreements were resolved through discussion or consultation with a third reviewer. Data were extracted independently by two reviewers using a standardized data extraction form. Extracted variables included author name, year of publication, country, study period, camel species, diagnostic method, sample size, number of positive animals, and reported prevalence. Where available, information on age, sex, season, body condition, and management system was also extracted for descriptive interpretation (22).

The primary outcome was study-level prevalence of *C. titillator* infestation. For studies reporting both direct infestation prevalence and serological positivity, only the direct infestation estimate based on larval detection or postmortem confirmation was included in the pooled meta-analysis to improve comparability across studies, whereas serological findings were retained for narrative interpretation (22, 23).

### 2.4 Quality assessment

The methodological quality and risk of bias of the included studies were assessed using the Joanna Briggs Institute (JBI) critical appraisal checklist for prevalence studies (23). The checklist consists of nine methodological domains evaluating sampling strategy, sample representativeness, sample size adequacy, study setting, data analysis, validity of diagnostic methods, reliability of measurements, statistical analysis, and response rate. Each item was scored as ‘yes’, ‘no’, ‘unclear’, or ‘not applicable’. Studies scoring 7–9 were classified as low risk of bias (high quality), scores of 4–6 as moderate risk of bias, and scores ≤3 as high risk of bias (low quality). Two reviewers independently performed the assessment, and disagreements were resolved through discussion. The quality appraisal was used to describe the overall strength of the evidence and to inform interpretation of the pooled estimates.

### 2.5 Statistical analysis

Statistical analyses were performed in R using the *meta* package (24). Because prevalence data are proportions and substantial between-study variability was anticipated, pooled prevalence estimates and their 95% confidence intervals (CI) were calculated using a random-effects model, which is recommended for meta-analysis of prevalence data (25). To stabilize the variance of proportions, prevalence estimates were transformed using the Freeman–Tukey double-arcsine transformation before pooling and were back-transformed for presentation (26). Between-study heterogeneity was assessed using Cochran’s Q test and quantified using the I^2^ statistic, with values above 50% considered indicative of substantial heterogeneity (27).

Prespecified subgroup analyses were conducted according to geographical region (Africa, Middle East, Asia, and Australia), study period (1980–2000 vs. 2001–2025), and camel species (*C. dromedarius* vs. *C. bactrianus*). The temporal stratification was selected to allow comparison between earlier epidemiological reports and more recent studies reflecting changes in camel husbandry systems, diagnostic approaches, veterinary management practices, and epidemiological surveillance over time, while maintaining an adequate number of studies within each subgroup. Studies with NR (not reported) study periods were retained in the overall meta-analysis when sufficient prevalence data (number of positive cases and total examined) were available. For subgroup analysis by study period, studies with NR were categorized according to their year of publication.

Small-study effects and potential publication bias were assessed using visual inspection of funnel plots and Egger’s regression test, while acknowledging that funnel plot asymmetry in prevalence meta-analyses may also arise from genuine heterogeneity (28). Although substantial heterogeneity was anticipated, formal meta-regression analysis was not performed because several potentially important covariates, including age, management system, season, body condition, and diagnostic approach, were inconsistently reported across the included studies, resulting in insufficient comparable quantitative data for robust meta-regression modelling. A *p*-value of < 0.05 was considered statistically significant throughout the analysis. Heterogeneity was further explored using prespecified subgroup analyses and by examining study characteristics, including diagnostic methods, sampling approaches, and geographical variation.

## 3 Results

### 3.1 Study selection and characteristics

**Figure 1:**
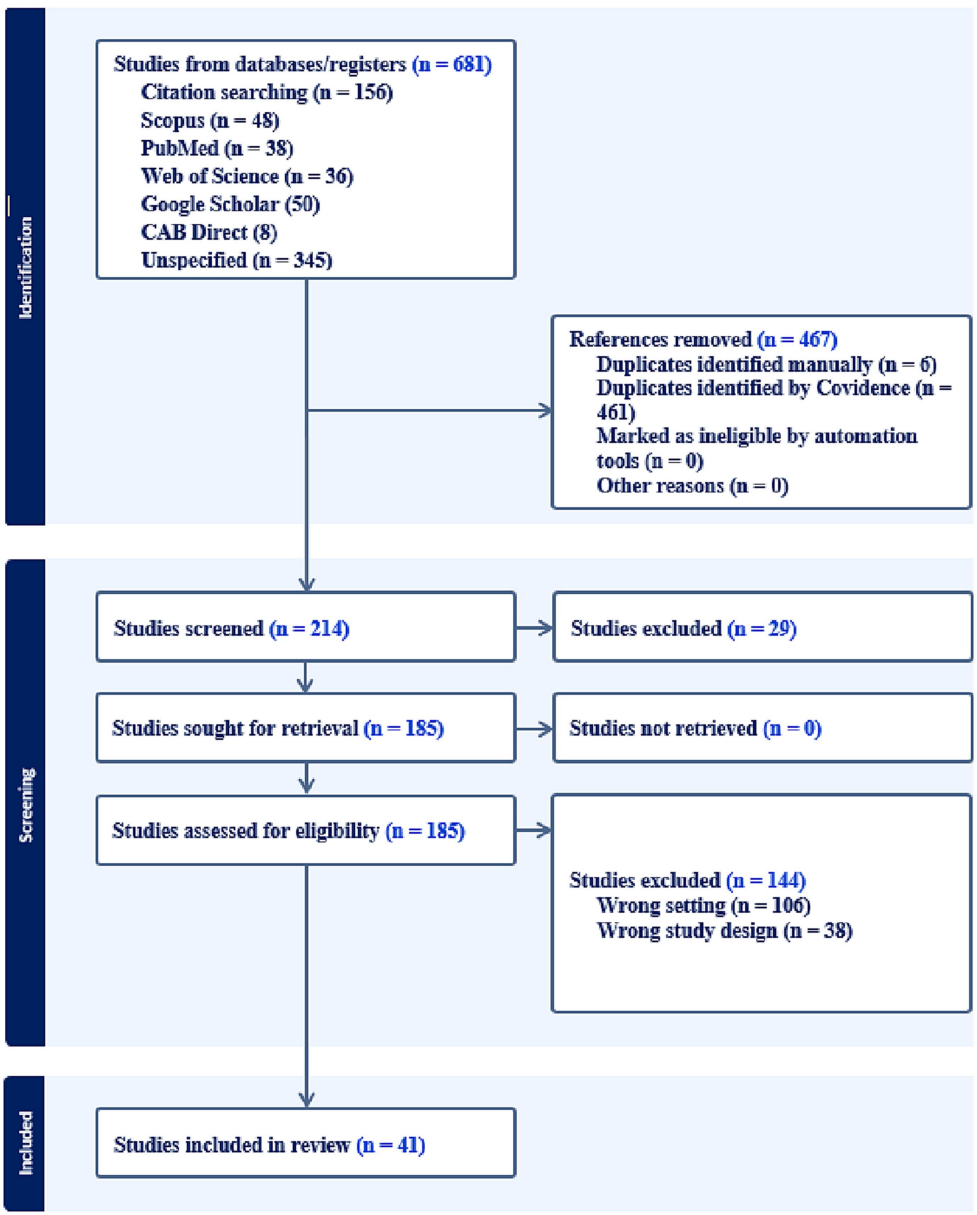
PRISMA 2020 flow diagram of study selection. A total of 831 records were identified, 214 studies were screened, 185 were assessed for eligibility, and 41 studies were included in the systematic review and meta-analysis.

The study selection process is summarized in Figure 1. After database searching, duplicate removal, and screening of titles and abstracts, potentially eligible articles underwent full-text assessment. A total of 41 studies met the inclusion criteria and were included in the systematic review and meta-analysis. These studies comprised 18,959 camels and were conducted across Africa, the Middle East, Asia, and Australia, involving both *C. dromedarius* and *C. bactrianus*. Table 1 summarizes the characteristics of all included studies, with five studies lacking information on the original study period coded as NR (not reported). These studies were nevertheless included in the overall meta-analysis because they provided extractable prevalence data, including the number of positive animals and the total examined. For studies reporting more than one prevalence measure, Table 1 specifies whether the extracted estimate used in the meta-analysis was based on direct/postmortem larval detection or another study-specific reference standard. Across the included studies, the reported prevalence of *C. titillator* infestation ranged from 26.7 to 100.0%, indicating substantial variability between study settings, host populations, and study periods.

**Table 1:** Summary of studies included in the meta-analysis reporting the prevalence of *C. titillator* in camels worldwide.

No	Author (Year)	Country	Species	Study period	Positive (n)	Total (n)	Prevalence (%)
1	Barton (38)	Australia	*Camelus dromedarius*	1988–1993	142	289	49.1
2	YunZhang et al. (39)	China	*Camelus bactrianus*	1995–1996	836	869	96.2
3	Yao et al. (1)	China	*Camelus bactrianus*	2019–2021	685	1,263	54.2
4	Khater et al. (34)	Egypt	*Camelus dromedarius*	2011–2012	100	240	41.7
5	Abu El Ezz et al. (7)	Egypt	*Camelus dromedarius*	2017	88	250	35.2
6	Hamed et al. (40)	Egypt	*Camelus dromedarius*	2019–2020	33	62	53.2
7	Attia et al. (9)	Egypt	*Camelus dromedarius*	2020	24	30	80.0
8	Hassanen and Abdel-Rahman (41)	Egypt	*Camelus dromedarius*	2021	32	120	26.7
9	Hassan et al. (20)	Egypt	*Camelus dromedarius*	2021-2022	24	38	63.2
10	Aboelsoued et al. (12)	Egypt	*Camelus dromedarius*	2022–2023	150	483	31.0
11	Bekele (31)	Ethiopia	*Camelus dromedarius*	1998–1999	558	778	71.7
12	Arabali and Gemeda (19)	Ethiopia	*Camelus dromedarius*	2013 – 2014	326	402	81.1
13	Regassa et al. (42)	Ethiopia	*Camelus dromedarius*	2014 – 2015	262	384	68.2
14	Abdilahi and Habtemichael (17)	Ethiopia	*Camelus dromedarius*	2015 – 2016	239	324	73.8
15	Kissi and Assen (43)	Ethiopia	*Camelus dromedarius*	2016 – 2017	276	334	82.6
16	Woldemeskel and Gumi (35)	Ethiopia	*Camelus dromedarius*	NR	546	546	100.0
17	Woldemeskel and Gumi (35)	Ethiopia	*Camelus dromedarius*	NR	121	121	100.0
18	Rajabloo (44)	Iran	*Camelus dromedarius*	1989	50	50	100
19	Oryan et al. (32)	Iran	*Camelus dromedarius*	2002–2005	771	1,328	58.1
20	Rad et al. (45)	Iran	*Camelus dromedarius*	2011–2012	138	286	48.3
21	Shamsi et al. (16)	Iran	*Camelus dromedarius*	2019–2021	339	870	39
22	Razi Jalali et al. (46)	Iran	*Camelus dromedarius*	NR	157	300	52.3
23	Radfar et al. (47)	Iran	*Camelus dromedarius*	NR	38	60	63.3
24	Shakerian et al. (48)	Iran	*Camelus dromedarius*	NR	310	384	80.7
25	Atiyah et al. (49)	Iraq	*Camelus dromedarius*	2008–2009	348	820	42.4
26	Al-jindeel et al. (8)	Iraq	*Camelus dromedarius*	2015–2016	352	864	40.7
27	Al-Kim and Al- Fatlawi (50)	Iraq	*Camelus dromedarius*	2021–2022	90	150	60.0
28	Essa et al. (18)	Iraq	*Camelus dromedarius*	2024	59	200	29.5
29	Al-Rawashdeh et al. (15)	Jordan	*Camelus dromedarius*	1996–1998	51	156	32.7
30	Al-Ani and Amr (51)	Jordan	*Camelus dromedarius*	1999–2000	45	97	46.4
31	Abdelrahman (33)	Libya	*Camelus dromedarius*	2007–2008	465	589	78.9
32	Desbordes and Ajogi (52)	Nigeria	*Camelus dromedarius*	1991-1992	367	528	69.5
33	Nwosu and Wachy (53)	Nigeria	*Camelus dromedarius*	1996	378	388	97.4
34	Mbaya et al. (54)	Nigeria	*Camelus dromedarius*	2010	200	234	85.5
35	Basu (55)	Nigeria	*Camelus dromedarius*	NR	229	250	91.6
36	Hussein et al. (3)	Saudi Arabia	*Camelus dromedarius*	1981	32	35	91.4
37	Hussein et al. (56)	Saudi Arabia	*Camelus dromedarius*	1981–1982	1,672	2,473	67.6
38	Fatani and Hilali (57)	Saudi Arabia	*Camelus dromedarius*	1991–1992	480	923	52.0
39	Al-Ahmed (58)	Saudi Arabia	*Camelus dromedarius*	1999–2000	350	860	41.0
40	Musa et al. (59)	Sudan	*Camelus dromedarius*	1983	44	44	100.0
41	Makin (60)	Sudan	*Camelus dromedarius*	2012–2013	300	537	55.9

NR indicates that the study period was not reported in the original article. A total of five studies lacked original study period information. Studies with unreported study periods were included in the overall meta-analysis when sufficient prevalence data (number of positive cases and total number examined) were available. For subgroup analysis by study period, studies with NR were categorized according to their year of publication.

### 3.2 Quality assessment

The methodological quality of the included studies was variable, with most studies classified as low to moderate risk of bias (Supplementary Table 1). Overall, 11 studies were categorized as low risk of bias (high methodological quality), 22 as moderate risk of bias, and 8 as high risk of bias (low methodological quality). Common methodological limitations included incomplete reporting of sampling frameworks, limited information on response rates, and the predominance of abattoir-based cross-sectional study designs, which may reduce representativeness of the broader camel population. These limitations should be considered when interpreting the pooled prevalence estimates.

### 3.3 Prevalence of *C. titillator* reported from the different countries of the world

**Figure 2:**
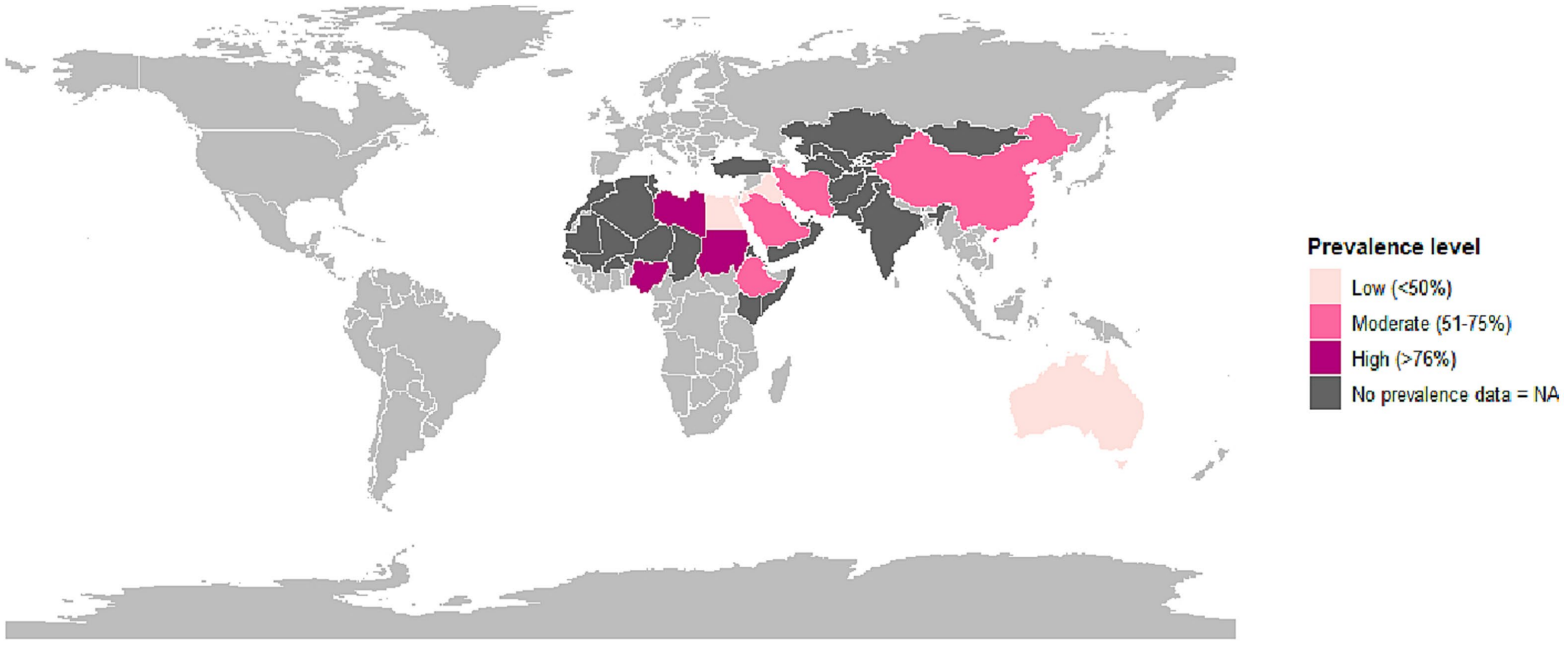
Global distribution of camel-rearing countries and prevalence of *C. titillator* infestation in camels. Countries are classified into low (<50%), moderate (51–75%), and high (>76%) prevalence categories. Camel-rearing countries without available prevalence data are shown in grey.

The global distribution of camel-rearing countries and available prevalence estimates of *C. titillator* infestation is presented in Figure 2. Camel production is primarily concentrated in arid and semi-arid regions across Africa, the Middle East, and Asia, with additional populations in Australia. Camel-rearing countries include Afghanistan, Algeria, Australia, Bahrain, Burkina Faso, Chad, China, Djibouti, Egypt, Eritrea, Ethiopia, India, Iran, Iraq, Jordan, Kazakhstan, Kenya, Kuwait, Kyrgyzstan, Libya, Mali, Mauritania, Mongolia, Morocco, Niger, Nigeria, Oman, Pakistan, Qatar, Saudi Arabia, Senegal, Somalia, Sudan, Tajikistan, Tunisia, Turkey, Turkmenistan, United Arab Emirates, Uzbekistan, and Yemen, as reported by global livestock distribution data from FAO and WOAH (6, 29, 30).

Countries with available prevalence data demonstrate moderate to very high levels of infestation. The highest prevalence values were observed in Nigeria (90%), Sudan (80%), and Libya (79%), indicating very high levels of infestation. High prevalence was also observed in Ethiopia (75%). Moderate prevalence levels were reported in Saudi Arabia (60%), China (54%), Iran (52%), Australia (49%), Iraq (42%), and Jordan (40%). In contrast, relatively lower prevalence was observed in Egypt (31%). Several camel-rearing countries lacked published prevalence estimates in the available literature and are shown in grey on the map. Overall, the spatial pattern indicates that *C. titillator* infestation is widely distributed across camel-rearing regions, with consistently moderate to very high prevalence in countries where data are available. The absence of data from multiple camel-rearing countries highlights important geographical gaps in epidemiological evidence.

### 3.4 Pooled prevalence of *C. titillator* worldwide

**Figure 3:**
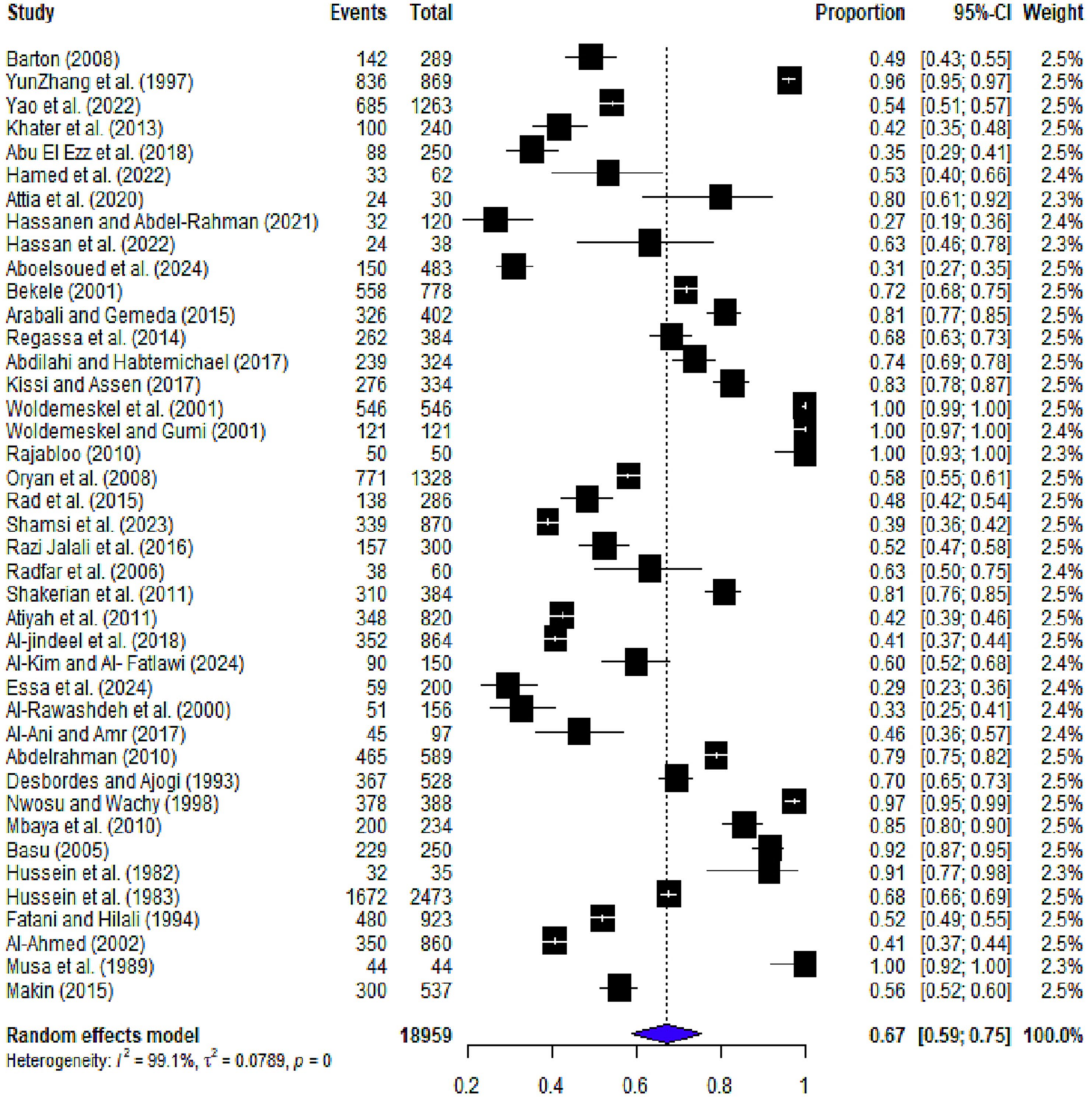
Forest plot of the pooled prevalence of *C. titillator* infestation in camels. Each square represents the prevalence estimate from an individual study, with horizontal lines indicating 95% confidence intervals (CIs). The size of each square reflects the weight of the study in the meta-analysis. The diamond represents the pooled prevalence estimated using a random-effects model. The vertical dashed line indicates the overall pooled estimate. High heterogeneity among studies is indicated by the I^2^ statistic.

The pooled global prevalence of *C. titillator* infestation in camels was estimated at 67% (95% CI: 59–75%) using a random-effects model (Figure 3). A total of 18,959 camels were included across all analysed studies. This finding indicates a high overall burden of infestation across the analyzed studies rather than a direct estimate of the global camel population. Given the extremely high heterogeneity (I^2^ = 99.1%), the pooled prevalence should be interpreted as a broad epidemiological summary across diverse ecological, geographical, and methodological settings rather than a single universally applicable prevalence estimate. Substantial variability was observed among studies. The heterogeneity analysis revealed a very high level of inconsistency (I^2^ = 99.1%, *p* < 0.001), suggesting that differences in prevalence estimates are likely influenced by variations in geographical regions, management systems, climatic conditions, diagnostic methods, and study periods. This degree of heterogeneity indicates that the pooled estimate summarizes highly diverse study populations and settings and should therefore be interpreted alongside subgroup findings and the observed range of prevalence values.

The forest plot (Figure 3) illustrates the distribution of individual study estimates and their corresponding 95% confidence intervals. While several studies reported moderate prevalence levels (40–70%), others demonstrated extremely high values approaching 100%, contributing to the observed heterogeneity. The size of each square reflects the statistical weight of the respective study, and the pooled estimate is represented by the diamond at the bottom of the plot. Overall, these findings indicate that *C. titillator* infestation is highly prevalent across included studies, although the considerable heterogeneity underscores the importance of subgroup analyses to better understand the sources of variation.

### 3.5 Geographical region-based subgroup analysis of pooled prevalence of *C. titillator*

**Figure 4:**
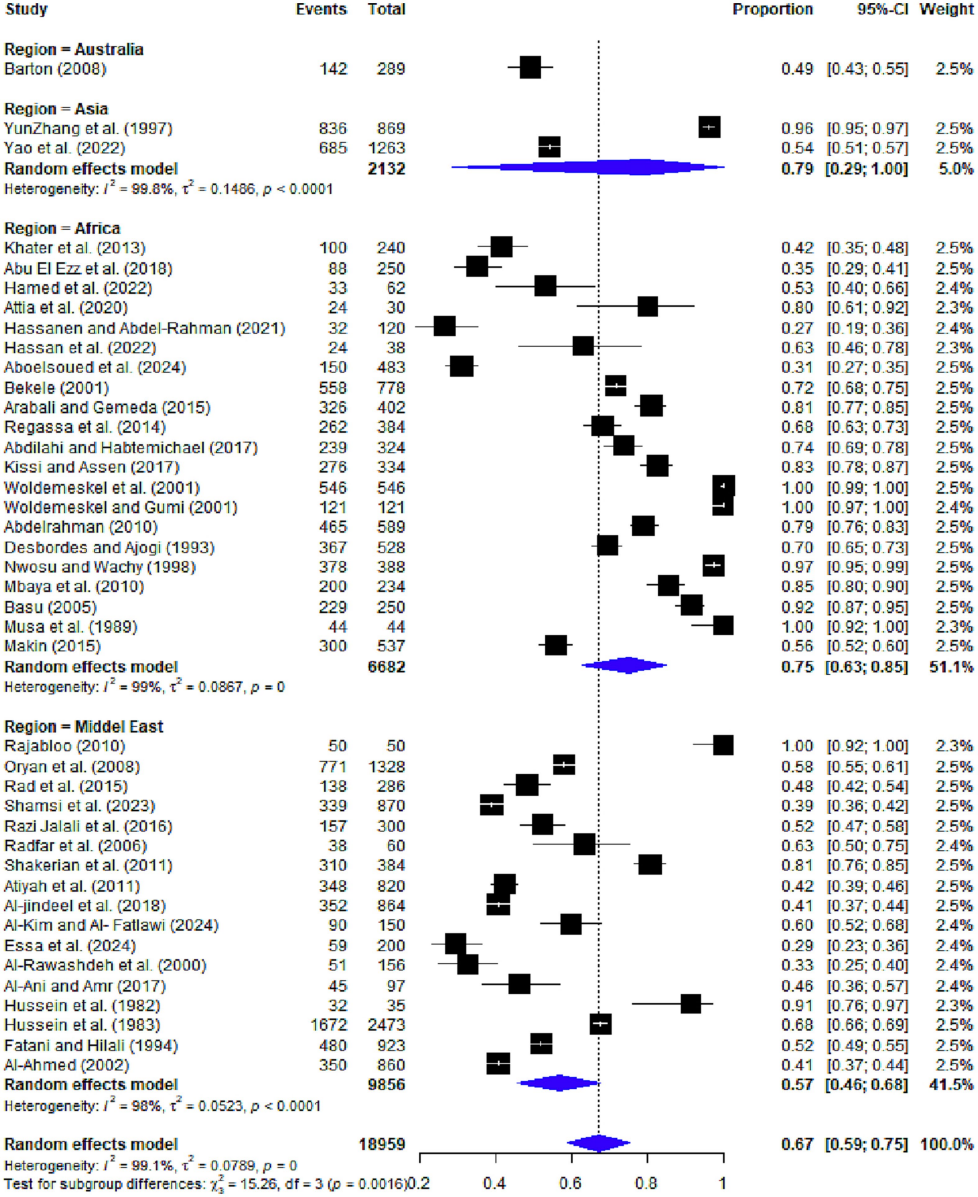
Forest plot of subgroup analysis by geographical region (Africa, Middle East, Asia, and Australia). Squares represent individual study estimates with 95% confidence intervals (CIs), and their sizes are proportional to study weights. Diamonds indicate pooled prevalence estimates for each region using a random-effects model. Statistical differences between subgroups are indicated by the test for subgroup differences.

Subgroup analysis based on geographical regions revealed statistically significant differences in the prevalence of *C. titillator* infestation (*p* = 0.0016) (Figure 4). Asia showed the highest pooled prevalence estimate at 79% (95% CI: 29–100). However, the very wide confidence interval indicates considerable uncertainty around this estimate, likely resulting from the limited number of available studies and substantial variability among the included study populations. Africa showed a consistently high and more precise pooled prevalence of 75% (95% CI: 63–85%), while the Middle East demonstrated a moderate pooled prevalence of 57% (95% CI: 46–68%). Australia was represented by a single study reporting a prevalence of 49%.

Despite stratification, high heterogeneity persisted within subgroups (I^2^ values remained elevated), suggesting that additional factors beyond geography, such as husbandry practices, climatic variation, diagnostic techniques, and study design, may contribute to the observed differences. Overall, these findings highlight that *C. titillator* infestation is widespread across all regions, but its prevalence varies significantly, with Africa and Asia showing the highest burden, underscoring the importance of region-specific control strategies.

### 3.6 Study period-based subgroup analysis of pooled prevalence of *C. titillator*

**Figure 5:**
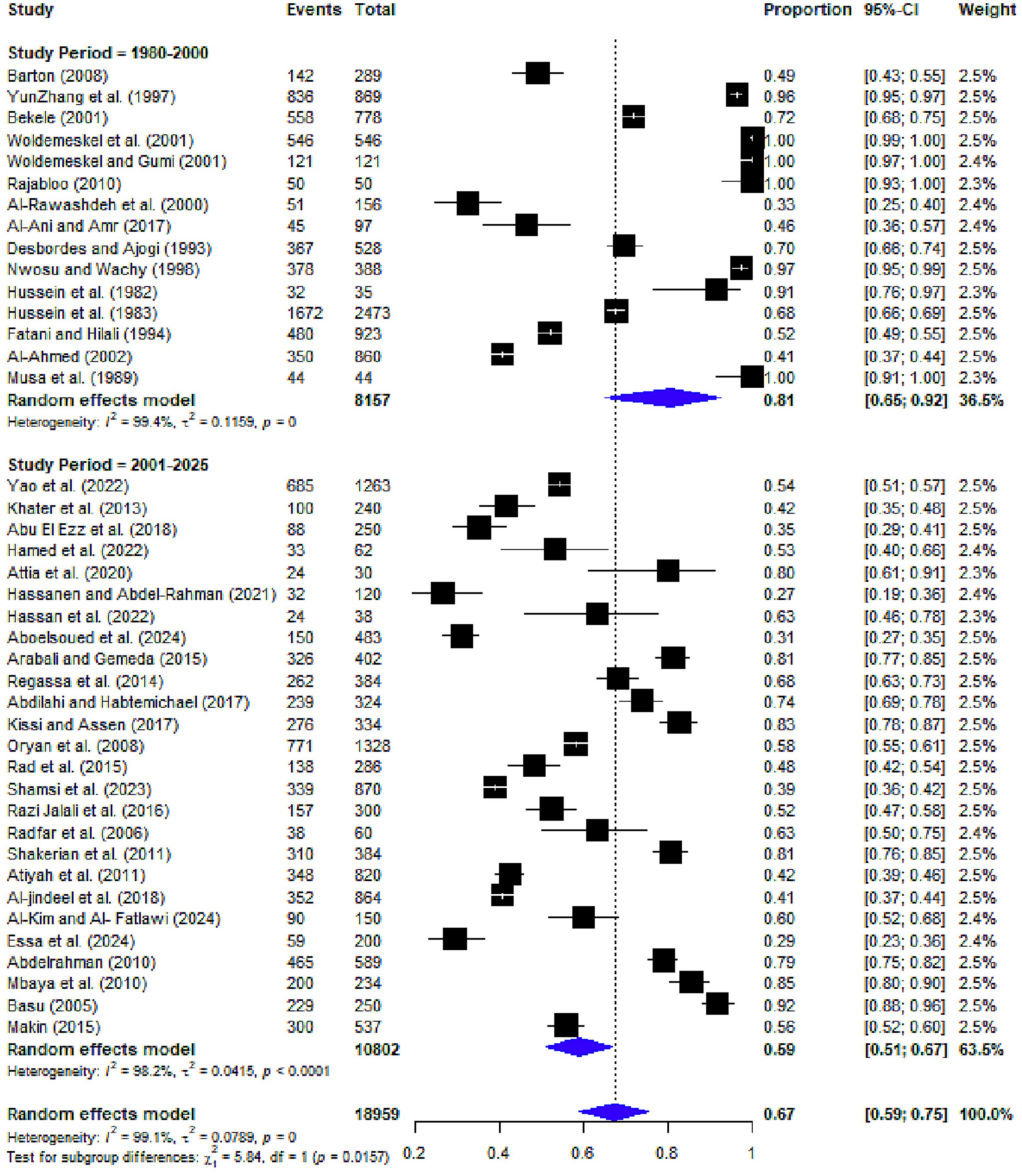
Forest plot of subgroup analysis by study period (1980–2000 vs. 2001–2025). Squares represent individual study estimates with 95% confidence intervals (CIs), with sizes proportional to study weights. Diamonds indicate pooled prevalence estimates for each subgroup.

Subgroup analysis based on study period demonstrated variation in the pooled prevalence of *C. titillator* infestation across time categories. The temporal stratification (1980–2000 vs. 2001–2025) was selected to allow comparison between earlier epidemiological reports and more recent studies reflecting changes in camel husbandry systems, veterinary management practices, diagnostic approaches, and epidemiological surveillance over time, while maintaining an adequate number of studies within each subgroup. Studies conducted between 1980 and 2000 showed a higher pooled prevalence of 78% (95% CI: 62–91%), whereas studies from 2001 to 2025 reported a comparatively lower prevalence of 61% (95% CI: 52–70%) (Figure 5). Although this pattern suggests a declining trend in prevalence over time, the difference between the two periods was not statistically significant (*p* = 0.0647). Therefore, the observed reduction should be interpreted with caution, as it may reflect random variation or differences in study characteristics rather than a true temporal decline.

High heterogeneity persisted within both subgroups (I^2^ > 98%), indicating that variability in prevalence estimates remains substantial even after stratification by study period. This suggests that other factors such as geographical distribution, management practices, diagnostic approaches, and environmental conditions continue to play a significant role in influencing prevalence estimates over time. Overall, while there is an indication of reduced prevalence in more recent studies, the lack of statistical significance highlights the need for further longitudinal and standardized investigations to confirm temporal trends.

### 3.7 Camel species-based subgroup analysis of pooled prevalence of *C. titillator*

**Figure 6:**
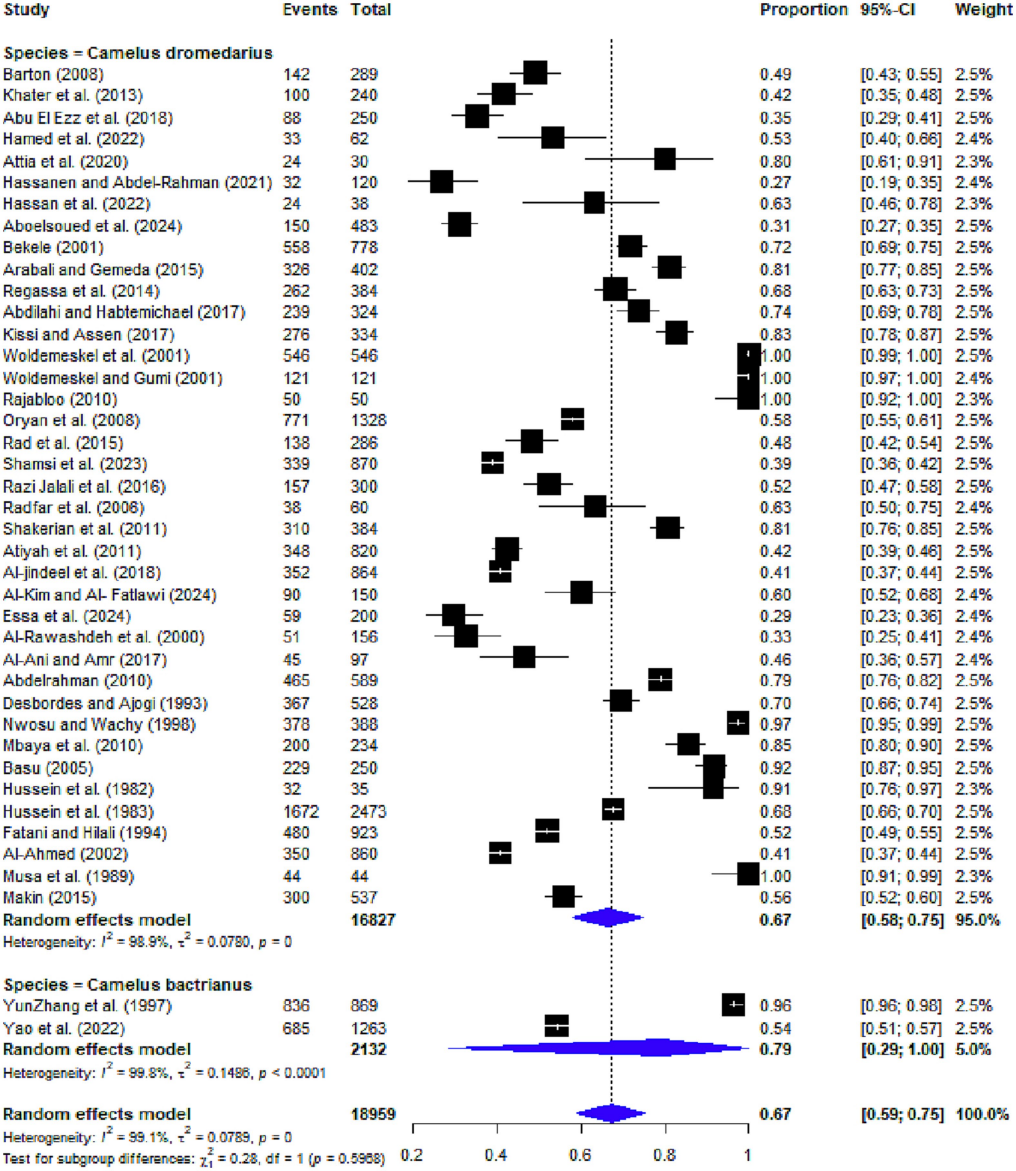
Forest plot of subgroup analysis by camel species (*C. dromedarius* and *C. bactrianus*). Squares represent individual study estimates with 95% confidence intervals (CIs), and diamonds indicate pooled estimates for each species using a random-effects model.

Subgroup analysis based on camel species demonstrated comparable prevalence estimates between *C. dromedarius* and *C. bactrianus*. The pooled prevalence for *C. dromedarius* was 67% (95% CI: 58–75%), based on the majority of included studies. In contrast, *C. bactrianus* showed a higher pooled estimate of 79% (95% CI: 29–100%); however, this estimate was accompanied by a wide confidence interval, indicating substantial uncertainty due to the limited number of available studies. The test for subgroup differences revealed no statistically significant difference between the two species (*p* = 0.597) (Figure 6), suggesting that susceptibility to *C. titillator* infestation is not strongly species dependent.

Despite this, high heterogeneity persisted within both subgroups (I^2^ ≈ 99%), indicating considerable variability in prevalence estimates regardless of species classification. This suggests that factors such as geographical location, environmental conditions, husbandry practices, and study methodologies may have a greater influence on infestation rates than host species alone. Overall, these findings indicate that *C. titillator* infestation affects both camel species at similarly high levels, although further studies on *C. bactrianus* are needed to improve the precision of estimates.

### 3.8 Exploration and interpretation of heterogeneity

Heterogeneity across included studies was extremely high (I^2^ = 99.1%, *p* < 0.001), indicating substantial variability in reported prevalence estimates beyond chance alone. In prevalence meta-analyses, such high heterogeneity is not unexpected when studies are conducted across broad geographical areas and under diverse ecological and methodological conditions. In the present review, prevalence estimates ranged from 26.7 to 100.0%, reflecting marked differences in infestation burden across study populations and settings.

Geographical variation appeared to be one of the major contributors to the spread and persistence of *C. titillator* infestation. Subgroup analysis demonstrated statistically significant regional differences (*p* = 0.0016), with higher pooled prevalence estimates observed in Asia and Africa compared with the Middle East. These differences are likely influenced by the combined effects of climate, husbandry practices, camel population density, and animal movement patterns. Arid and semi-arid environments characterized by high temperatures, seasonal fluctuations, and extensive pastoral production systems may provide favorable conditions for adult fly activity, larval development, and maintenance of the parasite life cycle. In addition, aggregation of camels around shared grazing and watering points may increase opportunities for transmission and repeated exposure to infective stages. Consequently, regional ecological suitability and herd management practices likely play central roles in shaping the epidemiology and geographical distribution of *C. titillator* infestation.

However, heterogeneity remained high within regional subgroups, indicating that geography alone did not fully account for the observed variation. Similarly, stratification by study period and camel species did not substantially reduce within-group heterogeneity, suggesting that additional unmeasured factors likely contributed to inconsistency in prevalence estimates. Narrative examination of study characteristics suggested several additional contributors to heterogeneity. Most included studies were abattoir-based cross-sectional investigations, while a smaller number used alternative sampling frameworks, which may affect representativeness of the source population. Diagnostic approaches also varied across studies, with most relying on direct postmortem larval detection, and fewer using immunological methods, which differ in sensitivity and in the type of infection status detected. Taken together, these findings suggest that the high heterogeneity likely reflects a combination of true epidemiological variation and methodological diversity across studies.

### 3.9 Publication bias and small-study effects

**Figure 7:**
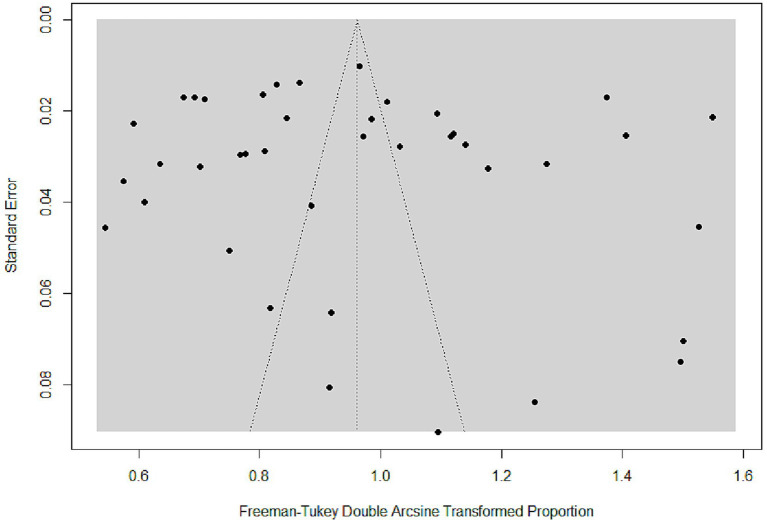
Funnel plot assessing potential publication bias in studies reporting the prevalence of *C. titillator* infestation in camels. Each point represents an individual study plotted against its standard error using Freeman–Tukey double arcsine transformed proportions. The vertical dashed line represents the pooled estimate, and the diagonal lines indicate the expected 95% confidence limits.

Potential publication bias was assessed using visual inspection of the funnel plot and Egger’s regression test. Visual inspection of the funnel plot (Figure 7) indicated slight asymmetry, suggesting potential small-study effects. However, in meta-analyses of prevalence, such asymmetry may arise from substantial between-study heterogeneity rather than true publication bias. The trim-and-fill analysis resulted in minimal adjustment to the distribution of studies and did not substantially alter the pooled prevalence estimate, indicating that the observed asymmetry is likely attributable to heterogeneity and methodological variability rather than systematic publication bias. Consistent with this interpretation, Egger’s regression test did not show statistically significant evidence of small-study effects (t = 0.69, *p* = 0.497).

## 4 Discussion

This systematic review and meta-analysis highlights the widespread epidemiological importance of *C. titillator* infestation across camel-rearing regions and emphasizes the substantial burden this parasite may impose on camel health, productivity, and animal welfare. The findings suggest that infestation remains a persistent parasitic challenge influenced by complex ecological, climatic, husbandry, and methodological factors. The marked heterogeneity observed among studies further indicates that infestation dynamics are likely context-dependent and shaped by regional environmental and management conditions. In addition, the limited availability of epidemiological data from several camel-rearing countries demonstrates that the current evidence base remains geographically uneven and incomplete. Collectively, these findings reinforce the veterinary and economic significance of *C. titillator* infestation and highlight the need for improved surveillance, standardized epidemiological investigations, and region-specific control strategies.

This review provides an important contribution to the understanding of *C. titillator* infestation by addressing the existing gap in epidemiological knowledge and integrating fragmented evidence from different regions into a global perspective. The pooled prevalence observed in the present review is generally consistent with previous regional epidemiological reports from Ethiopia, Sudan, Iran, and Iraq, where infestation rates frequently exceeded 40–80% (8, 16, 17). Similarly, broader parasitological reviews have identified *C. titillator* as one of the major myiasis-causing parasites affecting camel production systems in Africa and Asia (2, 5). Previous studies have largely been limited to specific countries or localized production systems, making it difficult to assess the overall burden and distribution of the disease. By synthesizing data across multiple camel-rearing regions, this study offers a more comprehensive epidemiological baseline and highlights both the widespread nature of infestations and the uneven distribution of available evidence. These findings are particularly significant given the increasing role of camels in food security, climate resilience, and livelihood support in arid and semi-arid regions, where reliable epidemiological information is essential for effective disease control and management.

Previous epidemiological studies have consistently demonstrated moderate to high prevalence of *C. titillator* infestation across camel-rearing regions using mainly postmortem larval detection methods. Reported prevalence estimates include 71.7% in Ethiopia (31), 58.1% in Iran (32), 79.0% in Libya (33), 41.7% in Egypt (34), 40.1% in Iraq (8), and 54.2% in China (1). Most studies diagnosed infestation through postmortem examination of the nasal passages, pharynx, and paranasal sinuses, whereas some investigations additionally employed immunological methods such as ELISA for detecting exposure in live animals (9, 20). Infection occurs when gravid female flies deposit first-stage larvae around the nostrils of camels, after which the larvae migrate into the nasal and pharyngeal cavities where they attach to mucosal tissues and undergo progressive developmental stages before being expelled for pupation in the external environment.

The extremely high heterogeneity observed in this meta-analysis (I^2^ = 99.1%) indicates that the prevalence of *C. titillator* infestation varies markedly across studies and settings. In systematic reviews of prevalence, such heterogeneity is common and often reflects the combined influence of biological, ecological, and methodological factors rather than statistical noise alone (25, 27). Therefore, the pooled prevalence of 67% should be interpreted as a summary estimate across highly diverse study contexts, rather than as a fixed prevalence value applicable to all camel populations or regions.

Several plausible factors may explain the magnitude of between-study variation. Geographical differences were one important source, as demonstrated by the significant subgroup effect by region. This is biologically plausible because the development, survival, and transmission dynamics of oestrid flies are strongly influenced by temperature, humidity, and seasonal conditions (1, 35), which vary substantially across Africa, the Middle East, Asia, and Australia. However, because heterogeneity remained high within regional strata, climatic variation alone does not fully explain the observed inconsistency. Differences in study design and source population likely contributed importantly. Most included studies were abattoir-based cross-sectional surveys, which may not accurately represent the wider camel population. Animals sent to slaughter may differ systematically from live herd populations with respect to age, health status, productivity, or body condition, thereby introducing selection bias and potentially distorting prevalence estimates.

Methodological diversity in diagnosis likely contributed substantially to variation in prevalence estimates. Most included studies relied on postmortem larval detection, whereas a smaller number used serological methods such as ELISA. Immunological studies have demonstrated higher apparent prevalence estimates using ELISA-based approaches compared with postmortem diagnosis, likely because serological assays detect both active and previous exposure (9, 12, 20). For example, ELISA-based studies reported prevalence estimates ranging from approximately 45.8 to 90%, whereas several postmortem-based studies reported prevalence values between 26.7 and 80%. Although postmortem examination is considered highly specific for larval detection, it may underestimate early or subclinical infections and does not capture infestation in live animal populations. In contrast, immunodiagnostic approaches such as ELISA offer higher sensitivity by detecting host immune responses, but they may overestimate active infection because of antibody persistence following previous exposure. Consequently, inconsistency between diagnostic approaches likely contributed to the wide variability in prevalence estimates and the persistently high heterogeneity observed across studies. However, because diagnostic methods were inconsistently reported and most studies relied on postmortem examination, formal quantitative subgroup comparison according to diagnostic method was not statistically robust.

In addition, management systems such as nomadic versus sedentary husbandry influence exposure risk, with higher prevalence observed in non-nomadic systems due to increased animal density, prolonged contact between animals, and greater environmental contamination (8, 36). In pastoral production systems, the aggregation of camels at shared watering and grazing points further facilitates parasite transmission by increasing opportunities for direct and indirect exposure to infective stages. These conditions promote sustained transmission cycles, particularly in environments where hygiene practices and parasite control measures are limited. Consequently, differences in herd management and animal movement patterns are likely to play a significant role in shaping the epidemiology of *C. titillator* infestation across regions.

No statistically significant difference was observed between camel species, with *C. dromedarius* showing a pooled prevalence of 67% and *C. bactrianus* showing a pooled estimate of 79% (*p* = 0.597). Although the point estimate was higher in *C. bactrianus*, the wide confidence interval indicates limited precision, suggesting that environmental and management factors are more influential than host species alone. Factors such as age, body condition, and husbandry practices further influence infestation risk, with higher prevalence observed in older and poorly conditioned animals, likely due to cumulative exposure and reduced immune competence, which may increase susceptibility to parasite establishment and persistence (1, 19).

*C. titillator* infestation causes irritation, inflammation, and tissue damage in the upper respiratory tract, leading to clinical signs such as nasal discharge, respiratory distress, reduced feed intake, and general weakness (16, 32). These pathological effects can result in decreased body condition, reduced milk yield, impaired fertility, and overall productivity losses (1, 5, 18). Chronic infestation may also induce immunomodulatory effects, including prolonged inflammatory responses, eosinophilic infiltration, cytokine-mediated tissue injury, oxidative stress, and potential suppression of host immune function, thereby increasing susceptibility to secondary infections (9, 10). Persistent larval antigenic stimulation and excretory-secretory products may contribute to chronic inflammatory dysregulation and progressive tissue pathology, which can exacerbate respiratory impairment and disease severity. Reproductive performance may be adversely affected due to chronic stress, poor nutritional status, and reduced physiological resilience, leading to decreased fertility and reproductive efficiency (5, 20).

The economic implications of infestation are substantial. In pastoral and agro-pastoral systems, where camels are central to livelihoods, reduced productivity, decreased milk and meat output, and increased management costs can significantly affect household income and food security (12, 16). Furthermore, the chronic nature of infestation raises important animal welfare concerns, particularly in heavily infested populations, where prolonged discomfort and respiratory impairment compromise overall well-being (7, 8). These combined effects highlight the significant veterinary and economic importance of *C. titillator* in camel-rearing regions (16, 17).

The findings of this review highlight the need for improved surveillance, diagnostic, and control strategies for *C. titillator* infestation. Future research should prioritize the development of sensitive and field-applicable diagnostic tools for early detection of infestation in live animals, particularly under resource-limited pastoral conditions. Longitudinal epidemiological studies investigating seasonal transmission dynamics, climatic influences, and risk factors are also needed. Standardized reporting frameworks for prevalence studies would improve comparability across regions and strengthen future meta-analyses. In addition, molecular characterization and phylogenetic analysis of *C. titillator* populations across different camel-rearing regions may improve understanding of parasite diversity and transmission dynamics. Further investigation into host immune responses, inflammatory biomarkers, and immunopathological mechanisms associated with chronic infestation is warranted. Finally, integrated parasite control strategies combining surveillance, strategic treatment, herd management, and environmental interventions should be evaluated under different production systems. Recent studies have also highlighted the potential larvicidal efficacy of *Mentha longifolia* essential oil against *C. titillator*, suggesting possible alternative control approaches with antioxidant and antiparasitic benefits (37).

## 5 Limitation

This review has several limitations. The very high heterogeneity among studies reduces the precision and generalizability of the pooled prevalence estimate. Most included studies were abattoir-based cross-sectional investigations, which may not fully represent the broader camel population and may have introduced selection bias. In addition, variability in diagnostic approaches, study designs, and reporting practices reduced comparability between studies. Important epidemiological modifiers such as age, sex, season, body condition, and management system were inconsistently reported and therefore could not be quantitatively assessed. Furthermore, inclusion of only English-language publications and the absence of eligible studies from several camel-rearing countries may have introduced language and geographical bias.

## 6 Conclusion

In conclusion, this study establishes a global epidemiological baseline and highlights key gaps in current evidence on *C. titillator*. These findings provide important insights that can inform stakeholders in developing standardized surveillance systems and region-specific control strategies for this neglected parasitic disease. The review showed that *C. titillator* infestation remains highly prevalent and widely distributed, likely driven by a complex interaction of ecological, biological, and management factors. Therefore, addressing the impact of *C. titillator* infestation is essential to enhance camel production systems and support the livelihoods of the communities who are depending on the camels for subsistence in arid and semi-arid environments of the world. Nevertheless, despite methodological variability and substantial heterogeneity among studies, the findings provide an important broad epidemiological summary of the global burden and distribution of *C. titillator* infestation in camels.

## Data Availability

The original contributions presented in the study are included in the article/Supplementary material, further inquiries can be directed to the corresponding author/s.
