# Supplementary material for: Global epidemiology of Cephalopina titillator infestation in camels: a systematic review and meta-analysis (1980–2025)
Source: Front Vet Sci. 2026 Jul 8;13:1863158. doi: 10.3389/fvets.2026.1863158 (PMC13388046; doi:10.3389/fvets.2026.1863158)
Supplement: Supplementary file 1 [file Table_1.DOCX]

**Supplementary Table 1**: Methodological quality assessment of included studies using the Joanna Briggs Institute (JBI) critical appraisal checklist for prevalence studies

| No | **Study** | **Q1** | **Q2** | **Q3** | **Q4** | **Q5** | **Q6** | **Q7** | **Q8** | **Q9** | **Total Y** | **Risk** |
| --- | --- | --- | --- | --- | --- | --- | --- | --- | --- | --- | --- | --- |
| 1 | Barton (20) | U | U | Y | Y | U | Y | Y | Y | U | 5 | Moderate |
| 2 | YunZhang et al. (21) | U | U | Y | Y | U | Y | Y | Y | U | 5 | Moderate |
| 3 | Yao et al. (1) | Y | Y | Y | Y | Y | Y | Y | Y | U | 8 | Low |
| 4 | Khater et al. (22) | U | U | Y | Y | U | Y | Y | Y | U | 5 | Moderate |
| 5 | Abu El Ezz et al. (4) | Y | U | Y | Y | Y | Y | Y | Y | U | 7 | Low |
| 6 | Hamed et al. (23) | U | U | U | Y | U | Y | Y | Y | U | 4 | Moderate |
| 7 | Attia et al. (6) | Y | U | N | Y | Y | Y | Y | Y | U | 6 | Moderate |
| 8 | Hassanen and Abdel-Rahman (24) | Y | U | Y | Y | Y | Y | Y | Y | U | 7 | Low |
| 9 | Hassan et al. (11) | Y | U | Y | Y | Y | Y | Y | Y | U | 7 | Low |
| 10 | Aboelsoued et al. (7) | Y | U | Y | Y | Y | Y | Y | Y | U | 7 | Low |
| 11 | Bekele (25) | U | U | Y | Y | U | Y | Y | Y | U | 5 | Moderate |
| 12 | Arabali and Gemeda (10) | U | U | Y | Y | U | Y | Y | Y | U | 5 | Moderate |
| 13 | Regassa et al. (26) | U | U | Y | Y | U | Y | Y | Y | U | 5 | Moderate |
| 14 | Abdilahi and Habtemichael (9) | U | U | Y | Y | U | Y | Y | Y | U | 5 | Moderate |
| 15 | Kissi and Assen (27) | U | U | Y | Y | U | Y | Y | Y | U | 5 | Moderate |
| 16 | Woldemeskel et al. (28) | U | U | Y | Y | U | Y | Y | Y | U | 5 | Moderate |
| 17 | Woldemeskel and Gumi (29) | U | U | U | Y | U | Y | Y | Y | U | 4 | Moderate |
| 18 | Rajabloo (30) | U | U | U | Y | U | Y | Y | Y | U | 4 | Moderate |
| 19 | Oryan et al. (31) | Y | U | Y | Y | Y | Y | Y | Y | U | 7 | Low |
| 20 | Rad et al. (32) | U | U | Y | Y | U | Y | Y | Y | U | 5 | Moderate |
| 21 | Shamsi et al. (2) | Y | Y | Y | Y | Y | Y | Y | Y | U | 8 | Low |
| 22 | Razi Jalali et al. (33) | U | U | Y | Y | U | Y | Y | Y | U | 5 | Moderate |
| 23 | Radfar et al. (34) | U | U | U | Y | U | Y | Y | Y | U | 4 | Moderate |
| 24 | Shakerian et al. (35) | U | U | Y | Y | U | Y | Y | Y | U | 5 | Moderate |
| 25 | Atiyah et al. (36) | U | U | Y | Y | U | Y | Y | Y | U | 5 | Moderate |
| 26 | Al-jindeel et al. (5) | Y | U | Y | Y | Y | Y | Y | Y | U | 7 | Low |
| 27 | Al-Kim and Al- Fatlawi (37) | Y | Y | U | Y | Y | Y | Y | Y | U | 7 | Low |
| 28 | Essa et al. (38) | Y | Y | Y | Y | Y | Y | Y | Y | U | 8 | Low |
| 29 | Al-Rawashdeh et al. (39) | U | U | Y | Y | U | Y | Y | Y | U | 5 | Moderate |
| 30 | Al-Ani and Amr (40) | U | U | U | Y | U | Y | Y | Y | U | 4 | Moderate |
| 31 | Abdelrahman (41) | Y | U | Y | Y | Y | Y | Y | Y | U | 7 | Low |
| 32 | Desbordes and Ajogi (42) | U | U | Y | Y | U | Y | Y | Y | U | 5 | Moderate |
| 33 | Nwosu and Wachy (43) | U | U | Y | Y | U | Y | Y | Y | U | 5 | Moderate |
| 34 | Mbaya et al. (44) | Y | U | Y | Y | Y | Y | Y | Y | U | 7 | Low |
| 35 | Basu (45) | U | U | Y | Y | U | Y | Y | Y | U | 5 | Moderate |
| 36 | Hussein et al. (46) | U | U | N | Y | U | Y | Y | Y | U | 4 | Moderate |
| 37 | Hussein et al. (47) | Y | U | Y | Y | Y | Y | Y | Y | U | 7 | Low |
| 38 | Fatani and Hilali (48) | Y | U | Y | Y | Y | Y | Y | Y | U | 7 | Low |
| 39 | Al-Ahmed (49) | Y | U | Y | Y | Y | Y | Y | Y | U | 7 | Low |
| 40 | Musa et al. (50) | U | U | N | Y | U | Y | Y | Y | U | 4 | Moderate |
| 41 | Makin (51) | U | U | Y | Y | U | Y | Y | Y | U | 5 | Moderate |

Y = Yes; N = No; U = Unclear. Methodological quality was assessed using the Joanna Briggs Institute (JBI) critical appraisal checklist for prevalence studies. Studies were categorized as low risk (7–9 “Yes”), modrate quality (4–6 “Yes”), or high risk (≤3 “Yes”).
